# Spontaneous Regression of Poorly Differentiated Carcinoma in the Transverse Colon with Deficient Mismatch Repair: A Case Report and Review

**DOI:** 10.70352/scrj.cr.26-0180

**Published:** 2026-06-19

**Authors:** Daisuke Takeyama, Fumiaki Mizuno, Takashi Suzuki, Atsushi Nakamura, Tohru James Harata, Seiji Chubachi

**Affiliations:** 1 Department of Surgery, Kurihara Central Hospital, Kurihara, Miyagi, Japan; 2 Department of Pathology, Tohoku University Hospital, Sendai, Miyagi, Japan

**Keywords:** spontaneous regression, deficient mismatch repair, high-frequency microsatellite instability, tumor-infiltrating lymphocytes

## Abstract

**INTRODUCTION:**

Spontaneous regression (SR) of colorectal cancer (CRC) is exceptionally rare. The SR of malignant tumors occurs in approximately 1 in 80000–100000 cases, and CRC accounts for <2% of all spontaneous malignancy regressions. Recent studies have suggested that immunological mechanisms, particularly those related to deficient mismatch repair (dMMR) and high-frequency microsatellite instability, may play an important role in such tumor regressions.

**CASE PRESENTATION:**

A 68-year-old woman was referred to our hospital after a positive fecal occult blood test. Colonoscopy revealed a 12-mm nonpolypoid lesion (IIa + IIc) in the transverse colon. Biopsy specimens showed poorly differentiated carcinoma without glandular formation or mucin production, accompanied by marked tumor-infiltrating lymphocytes (TILs). Immunohistochemistry was negative for CK20, CDX2, and neuroendocrine markers. The majority of TILs were CD3-positive lymphocytes. CT revealed no lymph node involvement or distant metastasis (cT1bN0M0). Laparoscopic partial colectomy of the transverse colon with D3 lymphadenectomy was performed 50 days after biopsy. Macroscopically, the resected specimen showed only a small scar-like lesion, and histological examination revealed no residual carcinoma. All dissected lymph nodes were tumor-free. Additional immunohistochemical analysis of the biopsy specimen showed loss of MLH1 and PMS2 expression, consistent with a dMMR status. These findings highlighted the possibility of medullary carcinoma, but a definitive diagnosis was not possible due to the limited biopsy samples. The postoperative course was uneventful, and no recurrence was observed during the 18 months of follow-up period without adjuvant therapy.

**CONCLUSIONS:**

We report an extremely rare case of SR of poorly differentiated CRC with dMMR and marked TILs. Enhanced tumor immunogenicity associated with dMMR and immune activation may contribute to CRC regression.

## Abbreviations

CRC
: colorectal cancer
DAMPs
: damage-associated molecular patterns
dMMR
: deficient mismatch repair
ICI
: immune checkpoint inhibitor
MSI-H
: high-frequency microsatellite instability
MSS
: microsatellite stable
pMMR
: proficient mismatch repair
SR
: spontaneous regression
TILs
: tumor-infiltrating lymphocytes

## INTRODUCTION

SR is a phenomenon in which a malignant tumor disappears completely or partially without special treatment.^1)^ It is estimated to occur in approximately 1 in 80000–100000 cancer cases.^2)^ SR in CRC accounts for less than 2% of these cases, making it an exceptionally rare event.^3)^

Previous studies have suggested several possible factors contributing to SR, including prolonged fever associated with sepsis, mechanical stimulation such as biopsy, primary tumor resection, stoma formation, and various psychological or genetic factors.^3)^ Evidence increasingly indicates that immunological mechanisms, particularly those associated with dMMR and MSI-H, play an important role in SR in CRC.^4,5)^

Poorly differentiated colorectal carcinoma is generally associated with a poor prognosis.^6)^ Differential diagnoses include poorly differentiated adenocarcinoma, neuroendocrine carcinoma, and medullary carcinoma.^7)^ Clinically, medullary carcinoma of the colon is observed more often in older women and is typically found in the right colon.^8)^ It presents with a low likelihood of lymph node metastasis and generally favorable outcomes, despite morphologic poor differentiation. Additionally, medullary carcinoma of the colon has a well-established association with dMMR.^8,9)^ Histologically, it is characterized by neoplastic cells with vesicular nuclei, prominent nucleoli, and abundant eosinophilic cytoplasm, arranged in solid sheets and exhibiting prominent intraepithelial lymphocyte infiltration.^10)^

We report a rare case of SR in CRC with dMMR. The tumor showed a poorly differentiated histology, and medullary carcinoma was suspected. A review of the relevant literature is provided alongside the case presentation.

## CASE PRESENTATION

**Fig. 1:**
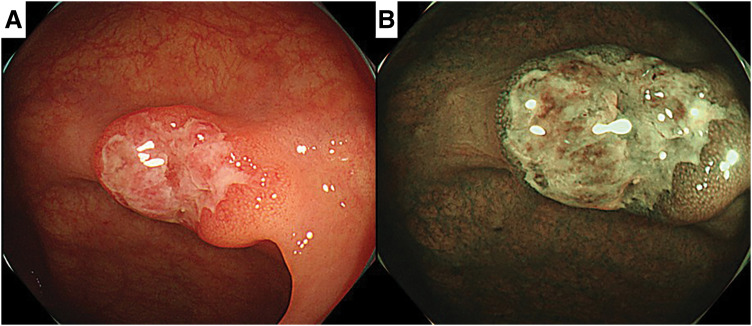
Colonoscopy image. Colonoscopy revealed a 12-mm nonpolypoid lesion (IIa + IIc) in the transverse colon, characterized by an elevated component with a central depression, an adherent white plaque, marked erythema, and an expansive appearance (**A**). Narrow-band imaging showed an absence of surface pattern and disrupted vessels (NICE classification Type 3) (**B**).

A 68-year-old woman underwent a routine medical check-up that revealed a positive fecal occult blood test, and was referred to our hospital for further evaluation. Colonoscopy revealed a 12-mm nonpolypoid lesion (IIa + IIc) in the transverse colon, characterized by an elevated component with a central depression, an adherent white plaque, marked erythema, and an expansive appearance (**Fig. 1A**). Narrow-band imaging showed an absence of surface pattern and disrupted vessels (NICE classification Type 3) (**Fig. 1B**). We also applied the e-T2 scoring system and confirmed that the lesion’s total score was below the 7-point threshold used to predict T2 invasion.^11)^ Endoscopic biopsy, tattooing, and clipping were then performed. Additionally, multiple colorectal polyps were identified. Her medical history included a caesarean delivery, and she was taking statin for dyslipidemia. Her family history was notable for lung cancer in her father and both biliary tract and lung cancer in her uncle.

**Fig. 2:**
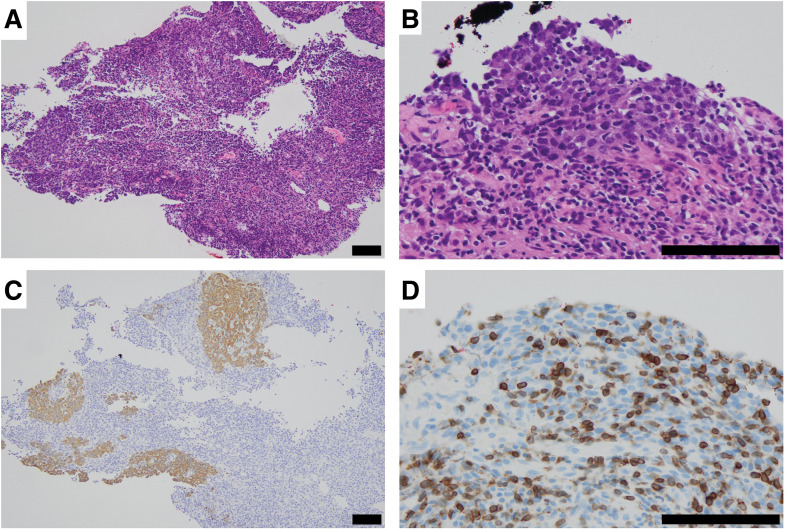
Histopathological findings of the biopsy specimen. Hematoxylin and eosin staining (**A**, **B**) and immunohistochemical staining for AE1/AE3 (**C**) indicated poorly differentiated carcinoma and prominent stromal inflammatory infiltrates. TILs were mostly positive for CD3 (**D**). Scale bar = 100 μm. TILs, tumor-infiltrating lymphocytes

Laboratory tests revealed no evidence of anemia. The CEA and CA19-9 levels were 2.4 ng/mL (reference <5.2 ng/mL) and 19.1 U/mL (reference <36.8 U/mL), respectively. Biopsy specimens demonstrated small nests of atypical cells lacking glandular differentiation or mucin production, accompanied by prominent stromal inflammatory infiltrates and abundant TILs (**Fig. 2A** and **2B**). Immunohistochemical staining was positive for AE1/AE3 (**Fig. 2C**) and p53, and negative for CK7, CK20, CDX2, synaptophysin, chromogranin A, and INSM1. Based on these findings, the lesion was diagnosed as poorly differentiated carcinoma. Most TILs were CD3-positive T lymphocytes (**Fig. 2D**), with only a few CD20-positive B cells. Barium enema revealed an arcuate deformity at the corresponding location. Contrast-enhanced CT showed no definitive primary mass, lymphadenopathy, or distant metastasis. According to the TNM classification, the clinical stage was cT1bN0M0 (cStage I).

**Fig. 3:**
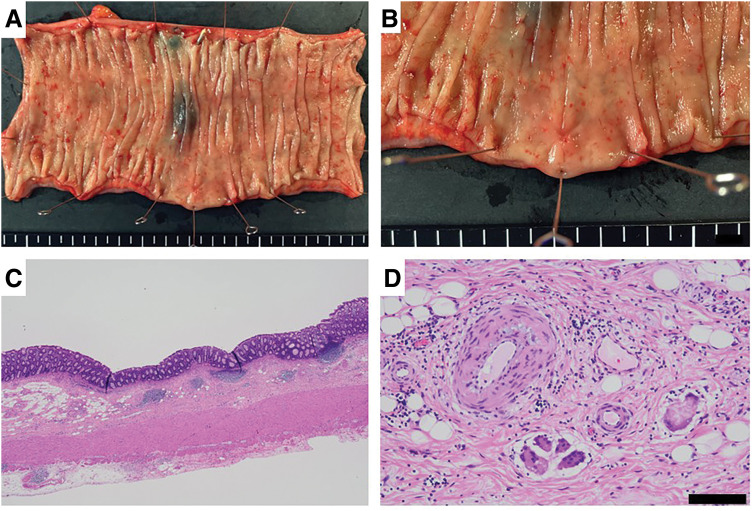
Gross and histological findings of the resected specimen. The resected specimen shows a 5-mm scar-like area (**A**, **B**). Histologically, no residual carcinoma cells were identified, with focal disruption of the muscularis mucosa, submucosal fibrosis (**C**), and foreign-body–type giant cells (**D**). Scale bar = 100 μm.

**Fig. 4:**
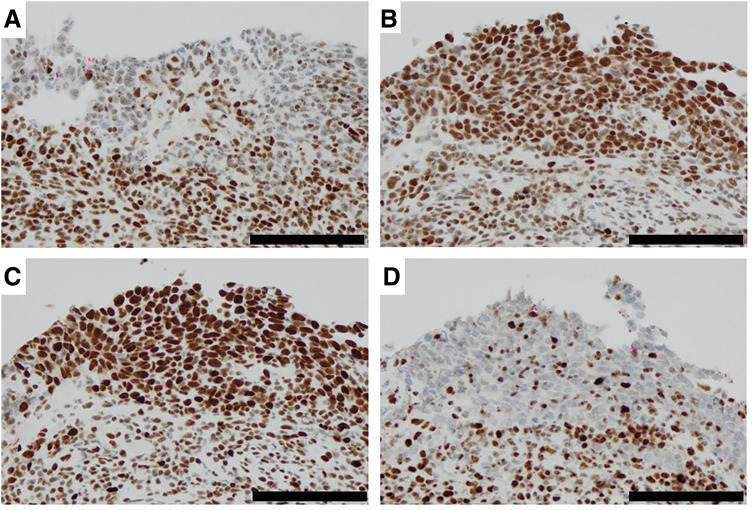
Immunohistochemical analysis of mismatch repair proteins. Immunohistochemical staining shows negative results for MLH1 (**A**) and PMS2 (**D**) and positive results for MSH2 (**B**) and MSH6 (**C**). Bar = 100 μm.

The patient was admitted to our hospital for surgery. There was no evidence of fever, infection, or alterations in medication use during the interval between the patient’s medical check-up and subsequent hospitalization. Fifty days after colonoscopy, laparoscopic partial resection of the transverse colon with D3 lymphadenectomy was performed. Gross examination of the resected specimen revealed flattening of the previously elevated lesion, which appeared as a 5-mm scar-like area (**Fig. 3A** and **3B**). Histological evaluation showed preserved mucosal architecture with focal disruption of the muscularis mucosae, submucosal fibrosis, and foreign-body–type giant cells with no residual carcinoma (**Fig. 3C** and **3D**). All the dissected lymph nodes were tumor-free. Because SR in CRC has been associated with dMMR, additional immunohistochemical analyses were performed on the biopsy samples. The lesion was negative for MLH1 and PMS2 and positive for MSH2 and MSH6, which was consistent with dMMR (**Fig. 4**). These findings suggested the possibility of medullary carcinoma; however, a definitive diagnosis could not be established.

The patient’s postoperative course was uneventful, and the patient was discharged on POD 10. Endoscopic mucosal resection of the remaining polyps confirmed the presence of serrated lesions. The patient has remained under surveillance for 18 months without adjuvant therapy, and no recurrence has been observed.

## DISCUSSION

SR occurrence in CRC is extremely rare. Abdelrazeq^3)^ reported 21 cases of spontaneously regressing CRC between 1900 and 2005. Similarly, Ohno et al.^12)^ summarized 22 cases of SR for primary colorectal tumors reported between 2000 and 2024. In our review of the literature, 29 reported cases of SR in CRC involving either primary tumors or metastatic lesions were identified between 2000 and 2025.^4,5,12–31)^ The characteristics of 30 cases, including the present case, are summarized in **Table 1**. The mean patient age was 72 years, and the male-to-female ratio was equal. Regression was observed in 25 primary tumors, 3 metastatic lesions, and 3 recurrent lesions.

**Table 1:** Reported cases of SR of the CRC

No.	Author	Age	Sex	Location	Histology	TILs	Clinical stage	Primary lesion			Metastatic lesion			Recurrent lesion	
								Regression degree	MMR status	MMR protein lost	Regression degree	MMR status	MMR protein lost	Regression degree	MMR status
1	Ikuta	60	M	R	Adenosquamous	NA	T3N3M1	No regression	NA		LN: no regression Liver: no regression	NA		Liver: partial	NA
2	Bir	86	F	Right side	Moderately	NA	T3N0M0	No regression	NA		NA	NA		LN: partial	NA
3	Sakamoto	80	M	R	Well	NA	T2N0M0	Complete	NA		NA	NA		NA	NA
4	Shimizu	80	M	T	Moderately	NA	T2N0M0	Complete	NA		NA	NA		NA	NA
5	Sekiguchi	69	F	A	Moderately	NA	T1N0M0	Complete	NA		NA	NA		NA	NA
6	Nakamura	60	M	R	Well	NA	T1N0M0	Partial	NA		NA	NA		NA	NA
7	Kihara	64	M	T	Moderately	NA	T2N0M0	Complete	NA		NA	NA		NA	NA
8	Chida	80	M	T	Poorly	TILs	T2N0M0	Complete	NA		NA	NA		NA	NA
9	Matsuki	72	F	A	Moderately	NA	T2N1M0	No regression	NA		LN: no regression	NA		Liver: complete	NA
10	Karakuchi	78	M	T	Poorly	TILs	T2N0M0	Complete	dMMR	MLH1/PMS2	NA	NA		NA	NA
11	Nishiura	67	F	T	Poorly	NA	T2N1M0	Complete	dMMR	MLH1/PMS2	LN: no regression	dMMR	MLH1/PMS2	NA	NA
12	Utsumi	78	M	A	Well	NA	T1N0M0	Complete	dMMR	PMS2	NA	NA		NA	NA
13	Utsumi	66	M	A	Moderately	NA	T1N0M0	Complete	dMMR	MLH1/PMS2	NA	NA		NA	NA
14	Utsumi	73	M	A	Moderately	NA	T1N0M0	Partial	dMMR	MLH1/PMS2	NA	NA		NA	NA
15	Yokota	76	F	T	Moderately	NA	T1N0M0	Complete	dMMR	MLH1/PMS2	NA	NA		NA	NA
16	Yokota	64	F	C	Well	NA	T1N0M0	Complete	dMMR	MSH2/MSH6	NA	NA		NA	NA
17	Yokota	64	M	T	Moderately	NA	T2N0M0	Complete	dMMR	MLH1/PMS2	NA	NA		NA	NA
18	Zwart	59	F	C	Mucinous	NA	TXNXM1	No regression	dMMR	MLH1/PMS2	Liver: complete	dMMR	MLH1/PMS2/MSH6	NA	NA
19	Harata	76	F	T	Well	NA	T2N0M0	Complete	dMMR	MLH1/PMS2	NA	NA		NA	NA
20	Shuttleworth	78	F	A	Poorly	NA	T2N0M0	Complete	NA		NA	NA		NA	NA
21	Shuttleworth	86	F	C	Moderately	NA	T1N0M0	Complete	NA		NA	NA		NA	NA
22	Pau	82	F	Hepatic	Poorly	NA	TXNXM1	No regression	dMMR	MLH1/PMS2	Peritoneum: complete	dMMR	MLH1/PMS2	NA	NA
23	Ortigão	42	F	R	Adenocarcinoma	NA	T2N0M0	Complete	NA		NA	NA		NA	NA
24	Ohno	83	F	T	Moderately	NA	T3N1M0	Complete	dMMR	MLH1/PMS2	NA	NA		NA	NA
25	Nakano	90	M	A	Moderately	NA	TXN1M0	A: complete T: no regression	A: dMMR T: pMMR	PMS2	LN: partial	NA		NA	NA
26	Watahiki	70	F	A	Well	TILs	T1N0M0	Complete	dMMR	MLH1/PMS2	LN: no regression	dMMR	MLH1/PMS2	NA	NA
27	Okano	54	M	T	Well	NA	T1N0M0	Complete	dMMR	MSH2/MLH6	NA	NA		NA	NA
28	Okano	75	M	A	Moderately	NA	T1N0M0	Complete	dMMR	MLH1/PMS2	NA	NA		NA	NA
29	Okano	84	M	R	Well	TILs	T1N0M0	Complete	pMMR		NA	NA		NA	NA
30	Present case	68	F	T	Poorly	TILs	T1N0M0	Complete	dMMR	MLH1/PMS2	NA	NA		NA	NA

References: 4,5,12–31.

A, ascending; C, cecum; CRC, colorectal cancer; dMMR, deficient MMR; MMR, mismatch repair; NA, not applicable; pMMR, proficient MMR; R, rectum; SR, spontaneous regression; TILs, tumor-infiltrating lymphocytes; T, transverse

Although the mechanisms underlying SR in malignant tumors remain unclear, several hypotheses have been proposed, including apoptosis, immune-mediated mechanisms, tumor microenvironmental factors, and infection.^32,33)^ In malignant melanoma, in which SR is relatively common, the prevailing hypothesis is that a high tumor-associated antigen load enhances antitumor immune responses, ultimately leading to tumor regression.^34)^ A similar immunological mechanism has been suggested for SR in CRC. In 2017, Chida et al.^20)^ reported a case of transverse colon cancer with marked TILs in biopsy specimens that subsequently underwent SR. In 2019, Karakuchi et al.^4)^ reported the first case of a spontaneously regressing CRC with dMMR/MSI-H. Since then, multiple reports have suggested an association between the dMMR/MSI-H status and spontaneous tumor regression in CRC.

For the effective activation of host antitumor immunity, tumor cells must be readily recognized by the immune system. The MMR genes MLH1, MSH2, MSH6, and PMS2 encode proteins that detect and repair base mismatches and insertion–deletion loops during DNA replication. Mutations in these genes result in MMR deficiency, leading to impaired DNA repair and alterations in microsatellite length, known as MSI-H.^35)^ Although MSI-H drives carcinogenesis by inactivating tumor suppressors through frameshift mutations, it simultaneously generates a high burden of neoantigens that significantly enhance the tumor’s immunogenicity.^36)^ Therefore, dMMR/MSI-H CRCs are more immunogenic than pMMR/MSS tumors.

Among the reported cases of spontaneously regressing CRC, the MMR status of the primary tumor was evaluated in 18 cases. dMMR was observed in 17 cases, and pMMR was observed in only 1 case. Nakano et al.^29)^ reported a case of synchronous multiple CRCs in which the dMMR lesion regressed spontaneously, whereas the pMMR lesion did not. These findings suggest a strong association between the dMMR status and SR. Additionally, in our review, 25 cases were localized to the right-sided colon, a finding highly consistent with the known clinical profile of dMMR/MSI-H CRCs, which frequently arise in the proximal colon.^35)^

dMMR/MSI-H status is associated with increased tumor immunogenicity and marked lymphocytic infiltration.^37)^ When neoantigens are presented by antigen-presenting cells and recognized by T cells, an antitumor immune response is induced.^38)^ Activated immune responses recruit cytotoxic lymphocytes that directly eliminate tumor cells.^39)^ In a systematic review and meta-analysis by Wankhede et al.,^40)^ the combination of MSI-H status and high TIL density was associated with the most favorable prognosis in CRC. Importantly, high TIL density was found to be a favorable prognostic factor regardless of the MSI-H or MSS status. Among the reported cases of SR in CRC, TILs were identified in the biopsy specimens in 5 cases. Notably, 1 case of SR with detectable TILs despite pMMR status has also been reported.^31)^ These findings suggest that the dMMR/MSI-H status alone may not be sufficient for SR, and that additional factors enhancing tumor immunogenicity may be involved.

Potential triggers of immune activation have also been proposed, with biopsy-related tissue injury being the most frequently cited mechanism.^5,18,29–31)^ In addition, endoscopic tattooing, as performed in our case, represents another potential source of localized physical and inflammatory stimulation. Procedures such as biopsy and tattooing may enhance this immune response through the release of DAMPs from injured tissue.^29)^ DAMPs derived from dying tumor cells, including calreticulin, heat-shock proteins, ATP, HMGB1, type I interferons, and IL-1 family cytokines, activate dendritic cells and enhance T-cell responses to tumor antigens.^41)^ Discordance in SR between primary and metastatic lesions has also been reported.^22,25,27,30)^ In these reports, only the primary tumor or the metastatic lesion regressed spontaneously despite the dMMR status of both lesions. For example, Watahiki et al.^30)^ reported a case in which the primary lesion and metastatic lymph node had dMMR status, yet only the primary tumor regressed, while lymph node metastasis persisted. They suggested that biopsy-induced DAMP release may have contributed to selective regression, as the biopsy was performed only on the primary lesion. Notably, in the reports of discordant cases, all lesions that regressed later were biopsied. These observations suggest that immune activation via DAMPs from injured tissue may play an important role in triggering SR.

Other potential mechanisms underlying this discrepancy have also been proposed. Watahiki et al.^30)^ also suggested that intratumoral heterogeneity, including clonal evolution and differences in the immune microenvironments of metastatic lesions, may influence tumor behavior. Zwart et al.^25)^ reported that metastatic lesions harbored an additional MSH6 mutation, suggesting an increased neoantigen burden and immunogenicity. Furthermore, several cases of SR have been reported only in recurrent lesions rather than at initial presentation.^13,14,21)^ Differences in the tumor microenvironment, intratumoral heterogeneity between primary and recurrent lesions, or immune-activating triggers during the interval leading to recurrence may have contributed to SR.

Another proposed mechanism of immune activation involves discontinuation of immunosuppressive therapy. Two cases have been reported in which patients receiving immunosuppressive agents for lung transplantation or rheumatologic diseases experienced SR in CRC after withdrawal of immunosuppressive therapy.^25,27)^ Additionally, infection-induced acute inflammation and fever have also been implicated as potential triggers for immune activation.^1,3,33)^

In the present case, the tumor exhibited the dMMR status and was therefore highly immunogenic. Abundant TILs were also observed, indicating the presence of immune effector cells actively targeting the tumor. There was no fever, infection, discontinuation, or initiation of medication between the medical check-up and surgery. Therefore, mechanical and inflammatory stimuli from the biopsy and tattooing were the most likely triggers for antitumor immune activation. However, the extremely dense inflammatory cell infiltration suggests that anti-tumor immunity may already have been activated by other factors at the time of biopsy.

In our review, 24 cases were staged as T1-2 and 23 cases as N0. This prevalence suggests that SR occurs more frequently in the early stages of tumor development. dMMR/MSI-H CRCs are characterized by high immunogenicity due to an abundance of neoantigens, which facilitates effective immune surveillance. Conversely, as tumors progress, they develop complex immune evasion mechanisms, including loss of HLA class I expression or upregulation of immune checkpoints such as PD-1/PD-L1, which can lead to metastasis and poor prognosis in advanced stages.^36)^ Therefore, SR may occur before these immune evasion mechanisms are fully established.

MMR status is an important biomarker for the efficacy of ICI therapy in CRC.^42)^ In recent years, the high therapeutic efficacy of neoadjuvant immunotherapy for dMMR CRC has been reported.^43,44)^ Specifically, the NICHE-2 trial reported that a short course of neoadjuvant nivolumab plus ipilimumab achieved a pathological complete response in 68% and a major pathological response in 95% of patients.^43)^ Given the shared background of dMMR and TILs, the dramatic tumor disappearance observed in this case may resemble the therapeutic effects of ICIs. It is conceivable that dysfunction of immune evasion mechanisms, including impairment of immune checkpoint function potentially induced by unidentified factors, may have permitted a sustained antitumor immune response, ultimately contributing to SR. In this modern oncological context, SR in dMMR cases can be viewed as a form of “natural” immunotherapy.

In summary, spontaneous tumor regression may arise from complex interactions among tumor immunogenicity, immune activation triggers, immune effector cells, and immune evasion mechanisms. High immunogenicity, as exemplified by dMMR/MSI-H status, may be more readily recognized by the immune system. Various triggers, including the release of DAMPs induced by biopsy or tattooing, withdrawal of immunosuppressive agents, and acute inflammatory events, may further enhance this response. Cytotoxic T lymphocytes, recognized as TILs, may exert antitumor effects. In addition, a potential dysfunction of tumor immune evasion mechanisms may facilitate an effective immune response.

The question arises whether all lesions were removed mechanically by biopsy. The tumor measured 12 mm, which is substantially larger than the tissue fragments typically obtained with biopsy forceps. In addition, regressive changes were observed in the submucosa of the resected specimen, including disruption of the muscularis mucosae, submucosal fibrosis, and foreign-body–type giant cells. These findings suggest prior submucosal involvement by cancer cells.^45)^ Taken together, we consider it unlikely that the lesion was mechanically removed by biopsy forceps.

Endoscopic resection was not considered in this case. Poorly differentiated histology is a high-risk factor for lymph node metastasis, and deep submucosal invasion was suspected, indicating a risk of incomplete resection. Therefore, surgical resection with lymphadenectomy was considered the most appropriate treatment. In addition, previous reports have demonstrated that lymph node metastases may persist even after SR of the primary lesion.^22,30)^ Even if SR had been observed after endoscopic resection in this case, surgical resection might still have been required, given the potential risk of lymph node metastasis.

Distinguishing medullary carcinoma from morphologically poorly differentiated CRC can be challenging.^7,46)^ In the present case, the biopsy specimen showed no glandular formation or mucin production and was initially diagnosed as a poorly differentiated carcinoma. Neuroendocrine carcinoma was excluded based on the absence of neuroendocrine markers. This case was characterized by an elderly female patient with a right-sided colonic lesion and a favorable clinical course, together with immunohistochemical findings of dMMR (loss of MLH1 and PMS2), prominent TILs, and CK20/CDX2 negativity. These findings highlighted the possibility of medullary carcinoma. Although loss of MLH1, CK20/CDX2 negativity, and calretinin positivity are useful immunohistochemical markers suggestive of medullary carcinoma,^47)^ morphological features remain the primary diagnostic criteria according to the World Health Organization Classification 6th Edition (unpublished data, online beta version). However, because the primary tumor in the present case underwent complete regression after biopsy, comprehensive histological evaluation of the entire lesion was not possible. Due to the limited sampling inherent to biopsy specimens, we could not definitively determine whether the entire tumor fulfilled the morphological criteria. Furthermore, interobserver reproducibility for the diagnosis of medullary carcinoma has been reported to be poor,^48)^ further complicating classification based on limited tissue samples.

It is also important to note that the dMMR/MSI-H profile with concurrent loss of CDX2 and CK20 is not specific to medullary carcinoma and may also be observed in a distinct subgroup of highly aggressive, non-medullary poorly differentiated carcinomas.^49)^ In contrast, the present case showed an exceptionally favorable clinical course, clearly differing from that typically seen in conventional aggressive poorly differentiated carcinomas. Given these diagnostic limitations and clinical findings, we consider the present tumor to represent a poorly differentiated carcinoma with a medullary phenotype. Biologically, the high immunogenicity of this medullary phenotype may have contributed to the strong immune response triggered by a minor stimulus such as biopsy, resulting in the dramatic SR observed in the resected specimen.

## CONCLUSIONS

We report a case of spontaneously regressed, poorly differentiated CRC with dMMR and marked TILs. Immunogenicity and immune responses associated with the dMMR/MSI-H status may play a key role in spontaneous tumor regression. Although rare, elucidating the mechanisms underlying spontaneous tumor regression may provide important insights for the development of more effective immunotherapeutic strategies.

## DECLARATIONS

### Availability of data and materials

The data generated or analyzed during this study are included in the published article.
